# Anatomical Mechanisms of Femoroacetabular Impingement and Its Role in the Progression to Hip Osteoarthritis: A Systematic Review

**DOI:** 10.7759/cureus.86461

**Published:** 2025-06-20

**Authors:** Sundip Charmode, Sudhir Shyam Kushwaha, Abhishek Kumar Mishra, Simmi Mehra

**Affiliations:** 1 Anatomy, All India Institute of Medical Sciences Rajkot, Rajkot, IND; 2 Orthopedics, All India Institute of Medical Sciences Gorakhpur, Gorakhpur, IND; 3 Orthopedics and Trauma, All India Institute of Medical Sciences Rajkot, Rajkot, IND

**Keywords:** acetabular version, cam, femoro-acetabular impingement, hip joint osteoarthritis, pincer, skeletal maturity, systematic review

## Abstract

Femoroacetabular impingement (FAI) causes hip pain because of unusual bone shapes at the area where the femur connects to the hip (cam-type) or at the hip socket (pincer-type). Due to the rarity of hip osteoarthritis, people in India often overlook FAI. Significant knowledge gaps exist in the underlying pathophysiology of FAI, impacting its progression. This study aims to look at the shape changes in the femoral head-neck junction in FAI and how these changes relate to the development of hip joint osteoarthritis. We chose original reviews, book chapters, and controlled trials written in English about the causes, symptoms, and progression of FAI that were published between January 2015 and December 2024 from the PubMed/Medline and Scopus databases. Case reports, series, editorials, commentaries, abstracts, and preprints were excluded. The risk of bias in systematic reviews (ROBIS) tool was used to assess the risk of bias in selected studies, and a standardized data extraction checklist was used to obtain pertinent data for synthesis. This research protocol was prospectively registered in the International Prospective Register of Systematic Reviews (PROSPERO) under registration ID CRD42025638992. A PubMed and Medline search using the MeSH term “femoro-acetabular impingement” yielded 3315 publications. We recorded 327 articles after filtering for English, humans only, no preprints, and all ages. Based on the title and abstract, 39 articles were shortlisted after a thorough review by two investigators, who finally selected 30 articles (four systematic reviews, 18 original studies, six review articles, one clinical trial, and one book chapter) for analysis. FAI appears to be primarily mechanical rather than inflammatory, with radiographic findings often not correlating with clinical severity. As the skeleton matures, the angle of the acetabulum increases, and early closing of the triradiate cartilage leads to pincer-type FAI.

## Introduction and background

Femoroacetabular impingement (FAI) is an important cause of early hip joint osteoarthritis [1-4]. The presence of FAI (cam deformity) in the general population is estimated at 14-24%, typically presenting in adolescents and young adults before arthritis develops [5]. Its prevalence in European, Japanese, and South Asian populations is 8-15%, 0.6%, and 5%, respectively [6]. Its prevalence is significantly higher in athletes (55%), with cam deformity in 54.8% and pincer deformity in 49.5% of cases [7]. FAI entails aberrant femoroacetabular articulation during flexion, adduction, and internal rotation [5]. FAI causes hip pain due to abnormal bone shapes at the femoral head-neck junction (cam-type) and acetabulum (pincer-type). When the hip is moved in extreme ways or used repeatedly, the unusual shape of the bones can lead to repeated stress on the joint, causing injuries to the labrum and damage to the cartilage, which can eventually lead to early arthritis in the hip joint [7]. India often overlooks FAI due to the rarity of primary osteoarthritis of the hip in its population [8]. Recent studies in India found that up to 67% of normal hips showed signs of FAI in X-rays, with 47.5% having pincer-type impingement, 7.9% having cam-type impingement, and 10.8% showing mixed signs [8]. Further, it is observed that the people who have cam-type morphology do not have FAI syndrome and do not develop hip osteoarthritis and remain asymptomatic throughout life [9]. As a result of the existing confusion in the terminology of FAI, asymptomatic people with cam-type deformity may have undergone treatment for FAI syndrome. Significant knowledge gaps also exist in the treatment of the aforementioned syndrome [10]. Recently, three systematic reviews and three narrative review articles on FAI were done, but they only looked at how it is seen in medical images and how common it is in different groups of people (those with symptoms, those without, athletes, and the general public). None of these reviews explored the underlying basis of FAI or studied its progression to hip joint osteoarthritis specifically in the Indian context (Table 1). To address this lack of information, this systematic review will look into the anatomical and embryological reasons for FAI and find out why it is more commonly seen in X-rays among the Indian population without leading to hip joint osteoarthritis. We also aim to understand the connection between symptoms, clinical exam results, and X-ray results in patients with FAI.

**Table 1:** Conclusions from other systematic reviews FAI, femoroacetabular impingement

Author details and year of publication	Aim of the review study	Results and conclusions
Mascarenhas et al. (2016) [11]	The study aimed to ascertain the frequency of FAI radiographic signs in athletes, asymptomatic individuals, and symptomatic patients.	The variety of FAI was more common in symptomatic cases compared to asymptomatic cases.
Nepple et al. (2015) [12]	To explore the relationship between adolescent participation in sports and the development of cam deformity.	Males involved in high-level sports such as hockey, basketball, and probably soccer face a 1.9-8.0 times higher risk of developing physeal abnormalities at the anteroposterior head-neck junction, leading to cam deformity.
Jauregui et al. (2020) [13]	To estimate the prevalence of FAI in a non-arthritic population with hip pain.	FAI should be considered in 47-74% of patients with hip pain and no evidence of arthritis.

## Review

Methods

This research protocol was prospectively registered in the International Prospective Register of Systematic Reviews (PROSPERO) on January 15, 2025, under the ID CRD42025638992 to ensure transparency and adherence to established guidelines. The study followed the PRISMA 2020 guidelines for searching studies, selecting them, extracting data, analyzing data, and risk of bias assessment [14]. The institute funded the study through the first author’s learning resource allowance. We have provided the PRISMA 2020 checklist as supplementary material with this manuscript.

Eligibility criteria

We searched the records in the initial survey using the eligibility criteria mentioned in Table 2.

**Table 2:** Eligibility criteria FAI, femoroacetabular impingement; RCT, randomized controlled trial

S. no	Inclusion criteria	Exclusion criteria
1	Complete original articles, review articles, book chapters, and RCTs published from January 1, 2015, till December 31, 2024	Case reports, case series, editorials, commentaries, letter to the editor, and preprints published before January 1, 2015, and after December 31, 2024
2	Publications in the English language	Publications in a non-English language
3	Publications focusing on the etiology, presentation, and progression of FAI	Publications focusing on diagnostic assessments and treatment strategies of FAI

Search strategy

We conducted a comprehensive and structured literature search across two medical databases, namely PubMed and Scopus, to identify eligible studies for this review. A Boolean search was conducted in the above-mentioned databases using the MeSH terms “femoro-acetabular impingement” or “FAI” and “hip osteoarthritis” or “hip joint osteoarthritis” for the duration between January 1, 2015, and December 31, 2024. We informed the institutional ethical committee and obtained an ethical waiver prior to the start of the review study. We have attached the ethical waiver letter as a supplementary file to this manuscript. The initial search led to 3315 publications.

Study selection

The initial 3315 recovered records were screened using automated filters in the PubMed and Scopus search engines, specifically for English language, free full text, only humans, and duplicates, as well as the inclusion and exclusion criteria. Three independent reviewers, SC, SK, and AM, conducted the screening of the titles using the eligibility criteria. If there were any discrepancies, we resolved them through conversation or by involving the fourth reviewer (SM). We downloaded and examined all the shortlisted full articles for relevant data using a standardized “data extraction checklist” that all four authors prepared. Table 3 displays the checklist.

**Table 3:** Checklist for data extraction FAI, femoroacetabular impingement

S. no	Data collected	Data ignored
1	The various types of FAI	The effectiveness of any new radiological investigation of FAI
2	The anatomical structures involved in the different types of FAI	The effectiveness of older and newer investigation methods of FAI
3	The anatomical basis of FAI	The effectiveness of older and newer treatment modalities of FAI
4	The embryological basis of FAI	The superiority of different treatment modalities for FAI
5	The presentation and progression to hip joint osteoarthritis	The biomarkers and their utility in early diagnosis and treatment of FAI
6	The hip joint movements are affected in FAI	The prevalence of different varieties of FAI in different populations
7	-	The impact of FAI on other pathologies of the hip joint, like the fracture of the femur neck
8	-	The association of another hip joint morphology with FAI

Data extraction

The data extraction checklist was pilot tested on 10 sample full articles by two authors (SC and AM) who thoroughly reviewed them separately. The study included details about the following: (i) the different kinds of FAI, (ii) the body parts involved in each type of FAI, (iii) the physical reasons for FAI, (iv) the developmental reasons for FAI, (v) the signs and symptoms of each type and how they can lead to hip joint osteoarthritis, and (vi) the hip joint movements and muscles impacted by the different types of FAI. Along with these data, information regarding the name of the first author, year of publication, country of origin, ethnicity of the studied population, and type of study design was also collected.

The information that was missing included the following: (i) how well new or old tests identify FAI, (ii) how effective the old and new treatments for FAI are, (iii) finding biomarkers and their role in early diagnosis and treatment of FAI, (iv) details on how common different types of FAI are in various groups of people, (v) how FAI impacts other hip joint issues like femoral neck fractures, and (vi) how different hip joint shapes are connected to FAI. Comprehensive data quality checks ensured accuracy and consistency during data recording.

Assessment of the level of evidence

The evidence level for each study was graded using the Grading of Recommendations, Assessment, Development, and Evaluation (GRADE) Working Group system [15]. One randomized controlled trial (RCT), four systematic reviews, six narrative/review articles, one book chapter, and 18 original observational studies from 2015 to 2024 were among the 30 chosen articles. The certainty of evidence was examined regarding how hip osteoarthritis develops, its relationship with clinical and radiological findings, and the causes of FAI related to growth and mechanics.

An RCT With One Participant

Initial grade: High Downgrades: Bias risk: Inadequate reporting of allocation concealment and randomization. Imprecision: Wide primary outcome confidence intervals and a small sample size (n = 82). Final score: Low certainty.

Systematic Reviews (n = 4; Approximately 5,400 Patients)

Initial grade: High Downgrades: Examples of inconsistencies include variable patient age, morphological definitions (such as alpha angle cut-offs of 50°-60°), and outcome metrics (I² > 60%). Indirectness: Development of hip osteoarthritis versus radiographic findings. Improvements: Some reviews indicated biological plausibility through dose-response associations, such as greater alpha angle and OA risk. Final score: Moderate certainty.

Initial Observations (n = 18; n ≈ 7,800)

Beginning grade: Low Downgrades: Bias: Selection bias was moderate in the majority of retrospective studies. Imprecision: Small subgroups (e.g., cam or pincer kinds) restricted generalizability in several studies. Improvements: Large effect size: Research has consistently shown that radiographic hip OA risk increases by 2.5-3.2 times when alpha angles are greater than 60°. Greater deformity was linked to earlier onset and quicker symptom progression in younger adults. Final score: Moderate to low certainty.

Narrative Reviews and Book Chapters (n = 7)

We did not assign a GRADE rating to these sources due to their lack of systematic methodology and reliance on expert opinion. They gave background information and explained ideas related to acetabular orientation and embryological development.

Risk of bias assessment

The risk of bias in each chosen review study was carefully evaluated by three independent reviewers, SC, SK, and AM, using the risk of bias in systematic review (ROBIS) tool that focuses on four domains [16]. We initially assessed the study’s relevance using phase 1 of the tool. The concerns of the review process of selected review studies were assessed using the study eligibility criteria, identification and selection of studies, data collection and study appraisal, and synthesis and findings. We settled any disagreements through discussion until we reached a consensus. We involved the fourth reviewer (SM) in cases where consensus was not possible. Below was a breakdown of bias risk: low, unclear, and severe.

Randomized Control Trial (n = 1)

The single RCT was prone to bias due to ambiguous random sequence generation, lack of allocation concealment, and lack of outcome assessor blinding. Performance and detection bias resulted from inadequate outcome data.

Four Systematic Reviews

There was moderate bias in all four systematic reviews. Two reviews lacked duplicate data extraction, and none offered comprehensive lists of excluded papers, despite strict inclusion criteria and search techniques. Inconsistent bias assessments in the primary research reviews raised doubts about the validity of the synthesis.

Observational Studies (n = 18)

Out of 18 observational studies: Because of their prospective designs, confounder adjustment, and explicit inclusion criteria, six studies had low bias.

Retrospective data collection, lack of blinding, and inadequate confounding variable control made eight studies moderately risky.

Small sample sizes, non-standardized FAI criteria, and radiographic versus clinical outcomes made four studies high-risk.

Narrative Reviews and Book Chapters (n = 7)

Since they provided conceptual rather than empirical support, ROBIS did not investigate them. Unfortunately, they are biased due to a lack of a systematic approach, transparent selection, and peer-reviewed grading.

Overall Outcomes

Five studies had a high bias, 12 had a moderate bias, and six had a low bias. The primary sources of bias were confounding, inadequate outcome data, selection bias, and a lack of FAI criteria. We took these limitations into account during the GRADE evidence grading and results synthesis.

Data analysis

We meticulously analyzed the extracted data to achieve the review objectives. We used the data to analyze the study design, including the age groups of patients, anatomical deformities recorded in the cases, and their commencement, along with their progression. We also utilized the data to examine the interventions given to the patients and their results. Our review study did not explore and assess the statistical analysis conducted in the reviewed articles. The observations and discussion of the selected studies were reviewed, and our comments were tabulated. We grouped the selected studies into two tables (Tables 4, 5).

**Table 4:** Articles describing the anatomical basis of FAI impingement FAI, femoroacetabular impingement; ROM, range of motion; FHNO, femoral head-neck offset; FH, femoral head; STS, sit to stand; ACAN, aggrecan; ADAMTS-4 = A Disintegrin and Metalloproteinase with Thrombospondin motifs-4

S. no	First author, year of publication, and country	Study design	Ethnicity of the population	Observations
1	Pierannunzii, 2019 [17]; Italy	Review article	Caucasian	The restricted hip ROM is compensated with coordinated enhanced lumbar curvature and pelvic tilt. Cam intrusion disrupts the fluid film.
2	Wylie et al., 2018 [18]; USA	Review article	American	Not having FHNO and FH asphericity causes movement issues, which result in damage to the cartilage at the chondrolabral junction while leaving the labrum unharmed. In the pincer-type, the labrum is affected by a crushing mechanism.
3	Samaan et al., 2017 [19]; USA	Comparative study	American	FAI patients increase hip demand and reduce knee load during STS tasks, accelerating hip cartilage degeneration and increasing the risk of osteoarthritis.
4	Chinzei et al., 2016 [20]; Japan	Comparative study	Asian	Larger alpha angles (cam-type FAI) were linked to higher levels of ACAN and ADAMT-4 genes, suggesting more inflammation and breakdown activity.
5	Kierkegaard et al., 2017 [21]; Denmark	Comparative study	Caucasian	FAI patients displayed reduced muscle strength (15-21% and 10-25%) in hip flexion and extension compared to the control group and the contralateral hip.
6	Kowalczuk et al., 2015 [22]; Canada	Systematic review	Caucasian	Cam-type deformity of FAI, especially with an elevated alpha angle, strongly predisposes patients to hip joint osteoarthritis, more so than the pincer-type impingement.
7	Ghaffari et al., 2018 [23]; USA	Review article	Caucasian	In FAI, bone deformities cause repeated hip joint impingement, resulting in soft tissue damage and faster progression to osteoarthritis. Occasionally, FAI can exist with normal osseous anatomy, especially in those having hyper joint mobility and excessive use.
8	Trisolino et al., 2020 [24]; Italy	Original article	Caucasian	25% of FAI patients displayed synovial mononuclear cell infiltration, which inversely correlates with HOOS pain, function, and total score change. All labral samples showed damage, 67% had calcium buildup, and the overall labral score is linked to more CD68+ synovial cells.
9	Tannast et al., 2015 [25]; Switzerland	Comparative study	Caucasian	They created standard measurements for the acetabulum and suggested using these for checking painful hips with AP pelvic X-rays, focusing on factors like LCEA, sharp angle, acetabular and extrusion index, and craniocaudal coverage.
10	Pettit et al., 2021 [26]; UK	Systematic review and meta-analysis	Caucasian	Caucasian Symptomatic FAI showed no overall inflammation based on sFLC levels, which also did not relate to how severe the disease was, indicating that it is mainly caused by mechanical issues.
11	Van Houcke et al., 2015 [27]; China and Belgium	Comparative study	Asian and Caucasian	Young asymptomatic Chinese and White subjects showed significant differences in hip anatomy.
12	Lawrenson et al., 2020 [28]; Australia	Comparative study	Caucasian	Ilio-capsularis may play a role in capsular retraction and enhanced hip joint protection during active and assisted hip flexion in FAIS.
13	Mimura et al., 2020 [29]; Japan	Observational study	Asian	In all radial slices, the β-angle was significantly smaller in the hips with symptomatic FAI than in the asymptomatic normal hips. The β-angle was the most useful parameter for diagnosing FAI, with the cutoff value of 53.6° at R60.
14	Mineta et al., 2016 [30]; Japan	Comparative study	Asian	Herniation pits primarily affected men in the Japanese cohort, and they were associated with abnormal femoral head morphology, specifically asphericity or small head-neck offsets.
15	Kierkegaard et al., 2017 [21]; Denmark	Comparative study	Caucasian	Patients with FAI demonstrate decreased hip flexion and extension strength when compared to (1) reference individuals and (2) their contralateral hip.
16	Kobayashi et al., 2016 [31]; Japan	Clinical trail	Asian	Accelerated local bone turnover was observed in painful hips, partly in FAI cases, and it was suggested that it has a significant role in its pathophysiology. It is also suggested that identifying it could facilitate diagnosis through imaging modalities.
17	Geoffrey Ng et al., 2016 [32]; Canada	Observational study	Caucasian	A reduced neck-shaft or medial proximal femoral angle may help predict hips at higher risk for early symptoms.
18	Moats et al., 2015 [33]; USA and UK	Comparative study	Caucasian	Cam-type deformity was seen to be absent in ancient humans, suggesting it arises from modern stresses. Further research on adolescent behavior may help prevent its development.

**Table 5:** Articles describing the developmental basis of FAI FAI, femoroacetabular impingement; ROM, range of motion; FHNO, femoral head-neck offset; FH, femoral head; STS, sit to stand; AV, acetabular version; TCC, triradiate cartilage closure

S. no	Authors, year of publication, and country	Study design	Ethnicity of the population	Observations
1	Pettit et al., 2021 [26]; UK	Systematic review and meta-analysis	Caucasian	Cartilage hypertrophy and epiphyseal extension precede CAM deformity. Fourteen years is the age of peak bone growth that marks the initiation of CAM deformity development. Sporting activity can increase the risk of CAM formation by two to eightfold.
2	Tak et al., 2015 [34]; Netherlands	Original article	Caucasian	The most susceptible period for the proximal femur is from age 12 onward, where sporting exposure leads to cam deformity.
3	Albers et al., 2017 [35]; Australia	Observational study	Caucasian	Increased acetabular version likely results from rotation during late maturation rather than differential growth. The steady ratio of acetabular depth to width and the coverage of the femoral head indicate that the triradiate cartilage may close too early, which could lead to pincer-type FAI.
4	Siebenrock et al., 2013 [36]; Switzerland	Original article	Caucasian	Cam-type deformity in athletes results from growth plate alteration and not reactive bone formation. High-level athletic activity before physeal closure demonstrated increased extension of the capital growth plate, contributing to cam formation. Greater physeal extension correlates positively with alpha angles.
5	Packer and Safran, 2015 [37]; USA	Review article	Caucasian	No strong evidence links FAI to genetics, but cam-type deformity is more common in high-level adolescent athletes. Cam deformity risk peaks near physeal closure, with few cases before 13 years and no risk after closure.
6	Laboudie and Beaulé, 2024 [38]; France and Canada	Book chapter	Caucasian	A cam lesion is defined as asphericity at the head-neck junction, which increases the risk of symptomatic impingement at the chondrolabral junction. Groin pain occurs with deep flexion and internal rotation, confirmed by the FADIR test.
7	Levy et al., 2015 [39]; USA	Original article	Caucasian	Subtle cam deformity significantly contributes to the pathoanatomy of symptomatic FAI in female patients.
8	Fortier et al., 2022 [40]; USA	Review article [updated]	Caucasian	All three morphological varieties of FAI exist in asymptomatic individuals, but intense athletic training and repetitive force contribute to abnormal bone growth.
9	van Klij et al., 2018 [41]; Netherlands	Review article	Caucasian	Cam morphology is linked to hip osteoarthritis development, whereas the association with pincer morphology remains unclear.
10	Kaymakoglu et al., 2021 [42]; Belgium	Original article	Caucasian	Cam and pincer morphology likely develop later in adolescence, after triradiate cartilage closure but before femoral physis closure.
11	Knapik et al., 2019 [43]; USA	Systematic review	Caucasian	Radiographic cam deformity is most common in soccer and hockey athletes. Males have significantly higher alpha angles than females, with no sex difference in diagnosis age.
12	Monckeberg et al., 2017 [44]; Chile	Original article	Caucasian	This study found no higher prevalence of FAI in adult soccer players compared to those with incomplete skeletal maturity, challenging prior assumptions. Elite soccer participation during skeletal immaturity does not appear to increase cam deformity risk in adulthood.

Results

Literature Search

**Figure 1:**
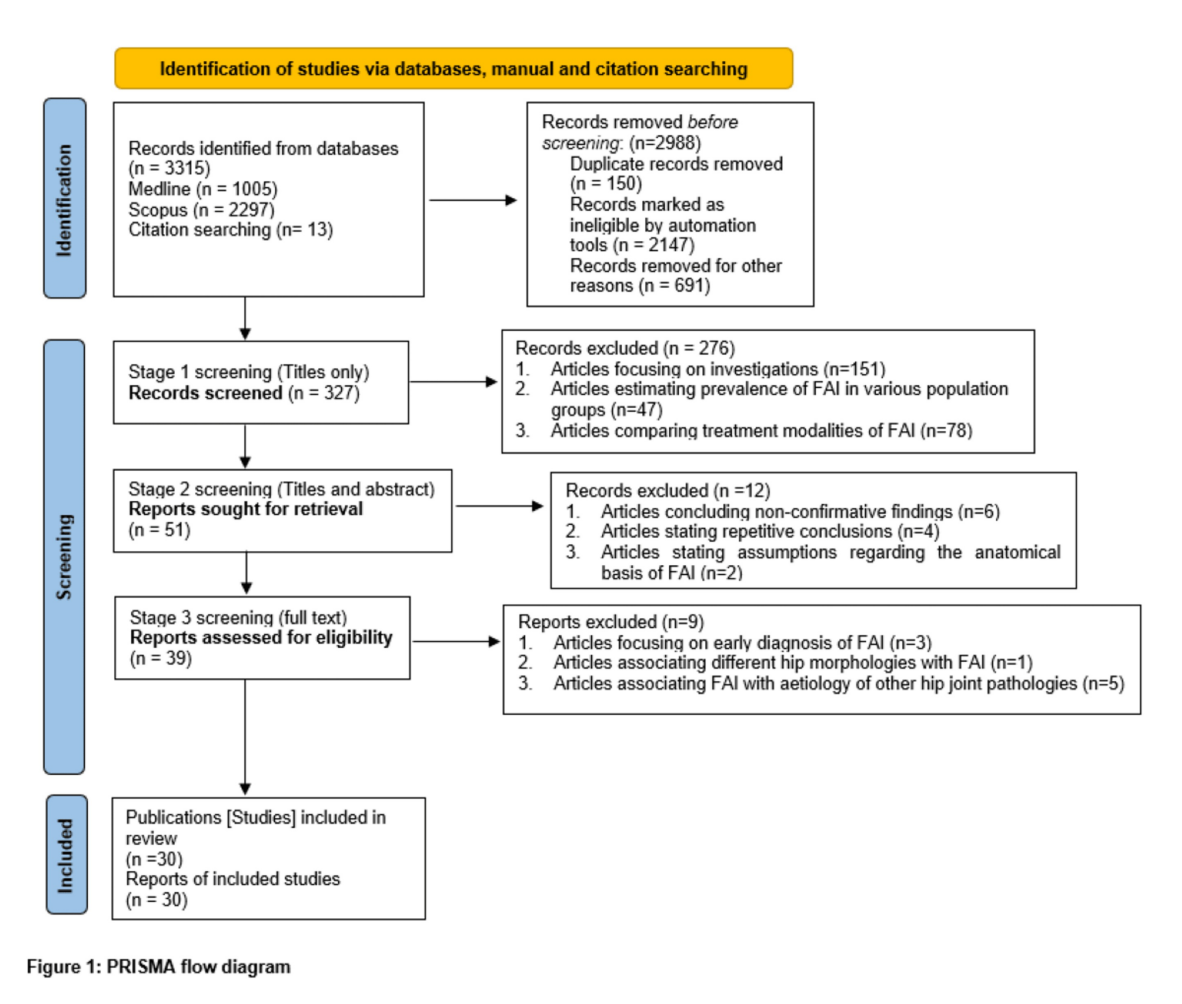
PRISMA flow diagram showing the step-by-step literature search

The initial online search using MeSH terms identified a total of 3315 records, including 1005 from Medline/PubMed, 2297 from Scopus databases, and an additional 13 through a citation search. After applying the automation filters to the search engine, 2147 records were eliminated, followed by 150 publications that were removed due to duplication, leaving behind 327. The stage 1 screening included a review of 327 publications by using the title only after application of eligibility criteria and was conducted by two reviewers separately (SC and AM), leading to 51 publications. The stage 2 screening involved carefully reviewing the titles and abstracts of these 51 publications by the same reviewers (SC and AM), which led to the exclusion of 12 articles for having unconfirmed findings and repeating conclusions based on assumptions, while 39 publications were selected for further review. These 39 shortlisted articles were reassessed thoroughly by reading the full text, and data were extracted using a data extraction checklist. In this stage 3 screening, a total of nine articles were excluded as they focused on early diagnosis, associating hip osteoarthritis with FAI, and associating FAI with the etiology of other hip joint pathologies. Ultimately, we selected 30 articles, including four systematic reviews, 18 original studies, six review articles, one clinical trial, and one book chapter. Figure 1 shows the step-by-step literature review conducted using the PRISMA flow diagram. We further reviewed the selected 30 publications and extracted relevant details based on the data extraction list. The selected articles were divided into two groups: the first group with 18 articles that looked at the anatomy related to FAI and the second group with 12 articles that examined the embryological basis of this condition. Table 4 presents the relevant details from 18 articles describing the anatomical basis of FAI. Table 5 shows important information from 12 articles that explain the embryological background of FAI. 

Discussion

Anatomical Basis of FAI

Pierannunzii (2019), Wylie et al. (2018), Siebenrock et al. (2013), and van Klij et al. (2018) found that cam deformity mostly harms the articular cartilage but does not affect the labrum, and this problem is significantly influenced by exercise, especially in teenagers [17,18,36,41]. These findings align with speculations of Murray and Duncan (1971) that a more than threefold rise in cam deformity might be triggered by intense adolescent exercises [45].

Pollard et al. (2010) found that siblings of patients with cam-type FAI are 2.8 times more likely to have the same deformity, while Packer and Safran (2015) disagreed, saying genetics don’t play a role [37,46].

Chinzei et al. (2016) observed that articular cartilage in FAI shows biologically higher inflammation and degeneration [20].

Talks et al. (2019) found that FAI patients did not have higher levels of sFLC, supporting the idea that FAI is a mechanical issue without overall inflammation in the body [47].

Embryological Basis of FAI

Kamenaga et al. (2023) linked problems with the ABAT gene, lower levels of DNMT3B, and inflammation from IL1β to the growth of cartilage cells and the breakdown of cartilage through MMP-13 and COL10A1 [48].

Grantham and Philippon (2019) found that the formation of cam lesions is linked to high-impact activities during the time when bones are still growing, highlighting that the period between when the skeleton is not fully developed and when the femoral head closes is crucial [49].

Wylie et al. (2018) stated that hip OA risk increased by 5% for every alpha angle degree >500 [18].

van Klij et al. (2018) found that having a cam deformity increases the risk of developing severe hip osteoarthritis within five years, with a 10.9% risk for an alpha angle greater than 60° and a 25% risk for an angle greater than 83°, while pincer deformity did not show this link [41].

Indian Scenario

FAI is a disorder in which clinical-radiological presentations can vary from near-normal to severe. Patients in the mild category, who are more common, may be unaware of their condition, which can worsen over time but can be managed with timely prophylactic measures, such as lifestyle changes. The mild category is often mistaken for other issues like fatigue, pain, intertrochanteric bursa syndrome, piriformis syndrome, labral injuries, worn-out hip joints, iliopsoas tendon tightness, tight adductor muscles, cysts near the hip, and avascular necrosis (AVN). The moderate category of patients is more likely to have persistent symptoms requiring repeated consultations. Those with severe categories are more likely to advance to progressive top hip OA. Pain subsides, followed by structural changes that settle over time, which retard the hip OA progression. 

Despite having radiologically positive FAI, it can be explained by the fact that in the Indian scenario, in routine daily activities, the biomechanical hip joint loading is on the knee joint and ankle joint, whereas the hip joint is bypassed. The bone density of the Indian population is higher than that of temperate countries due to year-round sunlight exposure, increased physical activity, and a greater proportion of stressful and strenuous activities, which contribute to a higher peak bone density. 

Limitations

The striking limitation of this study was that only two databases (PubMed and Scopus) were studied, and the work was done looking at the feasibility of the study. The second limitation was that only free full articles were included in the review, and many journals do not permit their articles to be downloaded freely.

Future implications

The main points found in this review will assist orthopedists in their everyday work by linking the signs and symptoms of FAI patients to the changes in their hip joint structure, which will help them offer better treatment.

## Conclusions

Cam-type FAI is strongly associated with the development of idiopathic hip osteoarthritis compared to pincer-type. Cam-type deformity is more common in males and Whites, suggesting a strong genetic basis, strongly influenced by high-impact exercises. FAI patients display reduced muscle strength (15-21% and 10-25%) in hip flexion and extension movements. Intensive adolescent sports activity promotes growth plate extension, strongly correlating with cam deformity development. Larger alpha angles (cam-type FAI) correlate positively with increased gene expression of ACAN and ADAMT-4.
